# On Gelsemium Sempervirens

**Published:** 1884-06

**Authors:** Charles Gilbert Davis

**Affiliations:** Chicago


### ARTICLE V.

### ON GELSEMIUM SEMPERVIRENS. By CHARLES GILBERT DAVIS, M.D., of Chicago.

The present advanced state of medical science is due in a great measure, if not entirely, to the recorded experience and observation of thinking men. If this be true, would it not be well if all physicians engaged in the active practice of their profession, made a special endeavor to observe more closely and record more accurately the various symptoms manifested by the action of medicine on the human system. A neglect of this on the part of many physicians has given rise to what we may properly term the "school of skepticism." They disbelieve entirely in the action of medicine to relieve disease, and often, while manifesting a sublime indifference to the appeals of the poor sufferer before them to be relieved, go into ecstasies over the pathology of the case, and are plethoric with theories in regard to the ultimate nerve-cells or molecules wherein the disturbing action arises.

Be it understood, I cast no reflections upon scientific investigation in these directions, but the true office of the physician is to prevent, cure, or alleviate disease, and in proportion as he fails in this, he fails to fulfil his mission.

To-night I lay before you my recorded experience in regard to the action of a remedy which for a number of years has been under my observation.

Gelsemium sempervirens, commonly known as the wild, yellow, or Carolina jessamine, is indigenous to the United States, and is found growing in great abundance in the rich soil on the borders of streams near the sea coast, from Virginia to Louisiana. It was first described in 1610 by John Parkinson, who cultivated it in his garden. It has a climbing stem; opposite, lanceolate, dark-green leaves; and axillary clusters of bell-shaped, yellow, fragrant flowers, the perfume of which has been compared to that of the wall-flower. The root, the part used in medicine, is fibrous, and owes its medicinal action to the presence of an alkaloid, gelsemia. According to Eberle, the central woody portion of the root does not contain any alkaloid, and therefore the bark really contains the active medicinal properties. I have been closely observing the action of gelsemium on the human system for a number of years, and am convinced there is no article of equal merit in the materia medica which has received so little notice, and concerning which so little is known. The object of the present paper is not so much to discuss the numerous theories concerning its mode of action, as merely to give a synopsis of these theories, and then to add my own testimony in regard to its therapeutic application, based upon personal experience and observation.

Some one has said that the medicinal action of this remedy was discovered by accident. A Southern gentleman who was sick with a fever, sent his colored servant into the garden to obtain the roots of a plant which he indicated, in order that he might prepare a decoction for himself and thus alleviate his disease. The servant made a mistake and obtained instead the roots of the yellow jessamine. The tea was prepared and drunk, however, and the patient breaking out into a profuse perspiration and recovering so soon from the fever, made inquiry as to what he had taken. He was so delighted with the result that he immediately recommended it to others who were similarly afflicted.

Gelsemium owes its medicinal action to the presence of an alkaloid, the active principle gelseminia, or gelsemia. A number of interesting experiments have been conducted by several observers in regard to the nature and action of this principle, the most conclusive of which are probably those by Professor T. G. Wormley. To him, undoubtedly, must be given the credit of having first isolated the alkaloid, and of having also demonstrated the presence of a new organic acid, which he designated gelseminic acid.

His process for obtaining the acid from the extract was as follows: "Concentrate the fluid extract on a water-bath to about one-eighth of its volume, and then add to the concentrated extract several times its volume in pure water, and allow the mixture to stand several hours, or at least until the supernatant liquid has become very nearly or altogether clear. By this treatment most of the resinous matter, held in solution by the alcohol originally present, will be separated. The mixture is then transferred to a filter, the solids well washed with water, and the filtrate thus obtained, together with the washings, concentrated on a water-bath to about the same volume the concentrated extract had prior to the addition of the pure water. The concentrated liquid, after filtration, if necessary, is acidulated with hydrochloric acid in the proportion of one drop of the pure acid for each fluid ounce of the fluid extract operated upon, then thoroughly agitated with about twice its volume of ether; after the liquids have completely separated, the ethereal fluid is decanted and the aqueous liquid finally washed with about its own volume of ether.

"On allowing the united ethereal liquids thus obtained to evaporate spontaneously, the gelseminic acid will be left chiefly in the form of nearly colorless groups of crystals. By this method sixteen ounces of the fluid extract of gelsemium yielded about two grains and a quarter of pure gelseminic acid. It is a colorless, odorless, mostly tasteless solid, crystallized in groups or tufts of delicate needles; has strongly acid properties neutralizing bases and uniting with them to form salts which are generally sparingly soluble in water.

"*Gelsemia* may be extracted from the concentrated extract from which gelseminic acid has been extracted by ether, by rendering the liquid slightly alkaline with potash, and then repeatedly agitating it with chloroform, which will dissolve the alkaloid together with more or less foreign matter. For this purpose about two volumes of chloroform may at first be employed, the operation repeated with a similar quantity of the fluid, when finally the alkaline solution is washed with about its own volume of the liquid. The chloroform employed for these extractions is collected in a dish and evaporated at a very moderate temperature, when it will leave a hard, gum-like, yellowish or brownish-yellow residue. This is treated with a small quantity of water and the mixture slightly acidulated with hydrochloric acid, which will dissolve the alkaloid together with more or less foreign matter. This solution is filtered, and the filtrate concentrated to about one-sixteenth the volume of the original fluid extract operated upon. On now treating the concentrated liquid with slight excess of caustic potash, the alkaloid will be precipitated in the form of a more or less white deposit. This is collected on a filter, washed with a small quantity of pure water, then allowed to dry at the ordinary temperature." When further purified by being dissolved in acidulated water and again precipitated by caustic potash, it will appear in the form of a very hard, brittle, transparent mass. When pulverized it forms a nearly or altogether colorless powder.

As to the amount of the alkaloid found in any given quantity of the fluid extract, the only official preparation used, it has not yet been definitely determined, but Wormley concluded from his experiments, approximately, that it existed to the extent of one grain of gelsemia to two and a half fluid ounces of the fluid extract.

*Physiological Actions.*—Investigations in regard to the action of the drug have been made by Ringer, Murrell, Ott, and Bartholow. Its various preparations have a narcotic odor and a bitter taste. The active principle, gelseminia, is so intensely bitter that its presence to the extent of one one-thousandth part by weight in solution can be readily detected. It is of a crystalloid nature, and when taken into the system is rapidly diffused into the blood. In regard to its action on the human system and also the lower animals, a number of interesting articles have been written. The most conclusive, however, are probably those experiments recorded by Prof. Bartholow. When administered in small quantities, sufficient to produce a sensible effect, it has a mild, soothing effect over the nervous system, manifested by mental quietude, a tendency to drooping of the eyelids, slight muscular relaxation, slowing of the pulse, and slight dilatation of the pupil; though Prof. Ringer says that this dilatation of the pupil is preceded by contraction. When the dose is much increased, the symptoms are all intensified; and we see manifested double vision, giddiness, pain in the forehead, increased paralysis of the levator palpebræ muscles, causing drooping of the eyelids; labored respiration from partial paralysis of the respiratory muscles; muscular weakness; and general sensibility much reduced. If the dose be still further increased, the symptoms are still more intensified, and we observe that the eyelids are entirely closed from complete paralysis of the levator palpebræ muscles; that the pupils are widely dilated, not responding to the action of the light; and that vision is entirely gone. Muscular paralysis is so complete that voluntary motion entirely ceases, respiration is labored, and the action of the heart becomes weak and intermittent. Consciousness is retained until near the period of death, when it is lost from carbonic acid poisoning, resulting from incomplete respiration. The action of the heart continues for some moments after respiration ceases. From these symptoms it is evident that the prime action of gelsemia is upon the nervous system, and its action is such as to warrant us in classifying it as a cerebro-spinal depressant, acting principally upon the motor centers. Some little discussion has arisen as to whether its primary action is upon the peripheral extremities of the motor nerves, or directly upon the nerve center. I believe that Prof. Ringer entertained at one time the opinion that its main action was upon the extremities of motor and sensory nerves; but more recently he has entertained the views of Prof. Bartholow, based upon a number of interesting experiments upon warm and cold-blooded animals, that the action is directly on the spinal cord. The order of this action is, however, upon the motor and sensory columns of the cord reversed in cold and warm-blooded animals. In the former, the first effect is on the sensory center; and in the latter, the motor centers are first impressed. In the *Phila. Med. Times*, Vol. V, will be found an interesting account of elaborate investigations carried on by Dr. Ott, in which he arrives at conclusions similar to those of Prof. Bartholow. He intimates that gelsemium reduces the pulse by lessening the irritability of the excito-motor ganglia of the heart; and lessens arterial pressure by diminishing cardiac irritability and vaso-motor tonus. He also, with Prof. Bartholow, concludes that it lowers the temperature. This last fact I consider one of great importance, having seen it verified in many febrile and inflammatory diseases. It would at first appear, from observing the action of gelsemium on the spinal cord, that it would be an efficient antidote to poisoning by strychnia; one excites to convulsions, while the other depresses to exhaustion. This theory, however, is not proven to be true by experiment. My own experiments on dogs have been conclusive in showing that it does not have the slightest influence in antidoting the effect of strychnia. On the contrary, I am inclined to believe that it facilitates or increases the action of strychnia. In order to arrive at a correct conclusion of the matter, I selected two dogs, weighing each within a fraction of ten pounds. To each of them I administered, at the same time, one-fifth of a grain of sulphate of strychnia hypodermically. Five minutes afterwards I gave one, to one-eighth of a grain of the alkaloid gelsemia in the form of a sulphate. In ten minutes from the time of the first injection, convulsions appeared in the dog that had received both strychnia and gelsemia; while the effects were not noticed on the other till the end of thirteen minutes. In thirty minutes, the animal that had received both poisons was dead, while the other lived forty minutes. It was noticeable in the experiment that in the animal receiving both the strychnia and gelsemia, the convulsions appeared earlier, were far more violent and continuous, and that death resulted ten minutes earlier. I have repeated this experiment a number of times with almost unvarying results. These experiments have not been as complete and satisfactory in this direction as I could wish, but sufficient evidence is obtained to convince me that gelsemium is in no way antidotal to the effects of strychnia; and I am much inclined to believe that, owing to the relaxing effects upon the spinal cord when gelsemium is previously administered, the conditions are rendered more favorable for the action of strychnia, and consequently its effects are intensified.

As antagonistic to, and incompatible with, the effects of gelsemium, we may rely upon alcoholic and diffusible stimulants. Tannic acid and the caustic alkalies may be considered as chemically incompatible with it. As an illustration of the action of alcoholic stimulants in removing the depressing symptoms resulting from the administration of large doses of gelsemium, I may mention a case that came under my observation some three years ago. J. B., aged 45, had been afflicted with muscular rheumatism for a number of years. The muscles of the right side of the neck, particularly the sterno-cleido-mastoideus and a portion of the platysma-myoides, had become so much affected that the chin was tied down to the breast, and mastication and deglutition were performed with great difficulty. A condition of complete torticollis had ensued. In this condition he applied to me to be at least temporarily relieved from the unpleasant and indeed painful muscular contraction. I immediately put him on five-drop doses of the fluid extract of gelsemium, to be taken three times a day, with instructions to increase the size of the dose one drop every day. He had reached a dose of twenty drops, and not seeing any of the usual effects of such a dose, and finding but little relaxation of the affected muscles, I requested him to change his druggist, thinking that perhaps the druggist with whom he dealt was not furnishing him with a reliable article. It is well known that there is occasionally a druggist who is no more infallible than the drugs he offers for sale to a too confiding public. Two hours after I had dismissed the patient from my office with this advice, he was brought back again by a policeman, who, finding him staggering on the street, had supposed him intoxicated. He showed to a very marked degree the effects of the remedy. The eyes were half closed, with inability to raise the upper lids, vision was obscured, and articulation was very indistinct, with general muscular relaxation manifested by a staggering gait, which so much resembled alcoholic intoxication as to well-nigh excuse the worthy officer, who supposed the patient to be under the influence of strong drink. A tablespoonful of brandy was immediately administered. In about fifteen minutes the patient spoke more distinctly, and expressed himself as much better. The same dose of the stimulant was repeated twice during an hour, at the expiration of which time the toxic symptoms had entirely disappeared, with the exception of a slight sense of drowsiness. This case not only serves to illustrate the antidotal action of alcoholic stimulants to the poisonous action of gelsemium, but also, what is true of many other of our fluid extracts, their frequent worthlessness as to medicinal action, when prepared by unscrupulous manufacturers.

*Therapy.*—Acting as it does on the great central nervous system, gelsemium must undoubtedly have a wide application therapeutically. In its native Southern region, it has for many years had an extensive reputation as a remedy of great value in the treatment of autumnal malarial fevers. While its efficacy in this direction may have been over-estimated, yet its beneficial effects in malarial fevers are such as to merit our careful consideration.

During several months' practice in the malarial regions of Missouri, I conducted a number of experiments to discover, if possible, the effects that gelsemium has upon malaria. After an observation of a number of years, during which time I have probably administered the remedy to many hundreds of cases, I have arrived at the following conclusions: First, gelsemium is not a direct antidote either chemically or physiologically to malaria; second, it does have a wonderful effect over the disease, owing to the relaxing influence it brings to bear upon the system, thereby increasing excretion, and assisting to eliminate morbific matters.

Third, I regard it as the great adjuvant of the salts of cinchona. In this respect, in my estimation, it nowhere has its equal; and in this direction, so far as malaria is concerned, lies its chief merit. In looking over my notebook, I find record of a case which came under my observation in the year 1873, in the city of St. Louis, and which fully illustrates this action. S. C., age 45 years; occupation, sign-painter; bilious temperament; sallow complexion; melancholy expression of countenance; said he had been afflicted with ague at intervals of a few weeks for some three years; had taken medicine from many physicians; had tried to take the various salts of cinchona, but the smallest amount produced such terrible cerebral symptoms that he now declared positively he would rather suffer with the disease than undergo the terrible agony produced by the quinine. He called upon me for treatment, and after he had given me a history of his case, I attempted to explain to him that I could administer the quinine in such a way as to prevent the usual unpleasant effects; but it was of no avail, he refused treatment. In a few days he was seized with a terrible paroxysm, simulating the congestive form. He, however, came out of this safely, but was again in forty-eight hours prostrated with another attack. A messenger came hurriedly for me, saying that Mr. C. was dying. I found him partially unconscious, in a congestive chill. After three hours of hard work, with diligent use of stimulants, external heat, etc., I succeeded in arousing him, and reaction began. During the febrile stage, I gave freely the fluid extract of gelsemium till I had reduced the temperature to the normal standard, and produced symptoms denoting the full effects of the remedy. To accomplish this, I gave for the first six hours three drops every hour, and afterward continued two drops an hour for twelve hours longer. At the end of this time, the patient being thoroughly under my control, or rather under the control of the medicine, and willing to take anything rather than risk dying in the third paroxysm, I immediately began the administration of sulphate of quinine, and gave ten grains every two hours till I had given 120 grains. I

then ceased and awaited results. It is needless for me to say there was no return of the disease. The patient was pleased; he had tried for years to take quinine, but could not endure its action. In the present instance, not a symptom of tinnitus aurium was manifested, and no other unpleasant symptom was noticeable. This is only one of a number of cases which now occur to my mind, in which I have seen gelsemium used to prepare the system for the action of quinine, where previously it was known to have a deleterious effect. That gelsemium is at all curative of malarial diseases, in the sense that quinine is, I do not believe; and yet I have observed that many so-called chronic cases, where the liver, kidneys, and entire excretory apparatus was torpid, were benefited or cured by this remedy. Its action in this direction, as I have before stated, I attribute to its influence over excretion, thereby enabling nature to throw off the disease through the natural channels.

*In convulsive diseases*, particularly those where the spasm is dependent upon reflex irritation, I know of no remedy that so satisfactorily fulfils the indications. In my own practice the most favorable results have been obtained in this direction in the various diseases of childhood in which convulsions are manifested. Children, as we all know, from a sensitive condition of the spinal centers and increased reflex irritability, are more liable to convulsions than adults. To quiet this irritation, knowing the action of gelsemium on the motor centers, we would naturally select it as the remedy *par excellence*. Such it has always proved itself to be in my hands. I have a number of cases recorded on my notebook to illustrate this action, but to mention one will be sufficient. Willie S., aged 8 months, was fed by his nurse for supper, milk and mashed potatoes. He had often been given the same diet for supper without deleterious results. On this occasion, however, as was afterwards discovered, the potatoes were not sound, and contained numerous hard lumps, which, when swallowed, the stomach was unable to digest. Consequently the child was thrown into convulsions. In one hour after this meal, spasms made their appearance. I was sent for, but did not reach the patient till about three hours after the beginning of the attack. During this time, according to the statements of the attendants, there had been twenty or more convulsions; this would be one in less than each ten minutes. As I entered the room the child was just relaxing from the last paroxysm. I immediately gave two drops of fluid extract, and in ten minutes two drops more. I then added ten drops of the fluid extract of gelsemium to four ounces of water, and ordered a teaspoonful of the mixture to be given every hour for twelve hours. No more convulsions appeared, and the next day the child was convalescent. Upon the whole, I think there is no remedy that has such a satisfactory effect on the nervous system of the child during the period of dentition, when the entire system is disturbed from irritation of the distal extremities of the fifth pair of nerves.

As a remedy for *meningeal inflammation*, both of the brain and spinal cord, I regard it as of great value. Some ten or twelve years ago, when cerebro-spinal meningitis prevailed more extensively over the country than at present, a number of cases came under my observation, all of which I treated with the remedy. I remember distinctly a case which serves to illustrate not only the efficacy of gelsemium in cerebro-spinal fever, but also the large amount of the remedy that may be borne when the nerve centers are irritated, or inflamed. I have no notes of this case, but write from memory. The patient was an intimate friend of mine, a medical student. When I was called to his bedside, the case had already progressed four days; his temperature was high; pulse rapid and small; tongue dry; mind wandering, and there was severe spinal tenderness. The slightest disturbance in the room, or jar of the bed, resulted in severe convulsions accompanied by opisthotonos. This occurred once or twice an hour. He had been given bromide of potassium to a large amount without apparent benefit. I immediately began the administration of gelsemium; and gave the fluid extract five drops an hour till I had given three doses, at which time another convulsion appeared. I then gave ten drops an hour; and in three hours there were slight symptons of another convulsion. I then administered twelve drops an hour; and gradually increased to fifteen, before there was entire relaxation and disappearance of all convulsive action. This dose was maintained for several hours, and then, as toxic symptoms began to be manifested, the dose was diminished.

He was kept under the influence of small doses for a week or more, and finally recovered. He was living when I last heard from him about three years ago, and was practicing medicine somewhere in the State of Michigan.

Gelsemium has been reported as used successfully in a number of cases of tetanus, but my own experience with it in this disease has been such as convinced me that it is of but little, if any use, in the tetanic condition. It has no effect, in itself, over epilepsy; but when used with the bromides it apparently intensifies the action of the latter. This action I have verified a number of times in cases of epilepsy confined to children. After using the bromides for some time without much apparent benefit, the gelsemium was added, and the desired result was more immediate and permanent. A number of observers have spoken of the remedy as one of great value in tic-douloureux, neuralgia of the fifth pair of nerves. My own experience with it in this direction is not encouraging. I have used it in a very few cases with apparent success, but my failures have far outnumbered my successful cases. How it ever gained a reputation for this form of neuralgia, I am at a loss to understand. It is possible that the successful cases were not those of tic-douleureux, but of neuralgia from cold, or from some other excentric causes producing congestion, which we may readily understand would be more liable to be relieved by this remedy.

Gelsemium may be used successfully in many of the acute inflammatory diseases. By lowering the temperature, and lessening the pulse rate, its natural tendency would be to lessen or retard the inflammatory action. I have used it in many cases of pneumonia and pleurisy with beneficial results. By its action over the heart it lessens the liability to pulmonary congestion, has a soothing effect over the respiratory function, and lessens the cough, thus giving rest to the affected organ, conditions which can be readily seen to be essential to a rapid recovery.

Gelsemium has a marked effect over the mucous surfaces. This I have seen frequently demonstrated by using it in gastro-intestinal catarrh, dysentery, and inflammatory conditions of the urinary organs. I have used it successfully in many cases to allay vesical irritability. Dr. Edward Mayer—*Hints on Specific*

*Medication*—recommends it highly in irritation of the bladder and posterior portion of the urethra. Where there was a marked irritability in this region, I have used it to successfully check nocturnal emissions; and found it a valuable remedy in chordee. Dr. Mayer also mentions this fact, that cases of enuresis, both infantile and senile, have been cured by gelsemium when belladonna had entirely failed.

Another application of gelsemium to which I wish to invite attention, came under my notice some twelve years ago. I was in Eastern Kansas, and my attention was called to quite a number of rattle-snake bites. The prairie rattle-snakes were numerous, and people were frequently bitten, many of the cases occurring among small children. The symptoms were always violent, and occasionally a death followed. Noticing the high inflammatory condition and frequent convulsions that followed the bite, I reasoned that gelsemium might prove an efficient antidote. I immediately began its administration, and must confess that no other remedy in my hands has ever yielded such results. The local inflammation and pain rapidly subsides, and convulsions cease in a short time after the first few doses. I had an opportunity to notice the effect of gelsemium in a number of these cases, and have also had favorable reports from several physicians to whom I suggested this plan of treatment. I not only gave the medicine internally, but had it applied locally. In these cases I regard its action as entirely physiological, and not chemical. The use of gelsemium in rattle-snake bites was first suggested to me by my father, Dr. Geo. W. Davis, of Foster, Bates county, Missouri.

*Diseases of Women.*—Another application of this remedy, and one which may be considered equal to, if not greater in importance than, all others, is that which relates to the pelvic disorders of women and their attendant nervous disturbances. This declaration may apply with equal force to both the acute and chronic states. The relaxing effect which it possesses over the uterus and its appendages, is something remarkable. In the first stage of labor, when the pains are of that peculiar short, sharp, and irritating nature, which is so exhausting to the patient and wearying to the patience of the physician, a few drops of the remedy will immediately change the features of the case, the pains assuming a more steady and expulsive nature. When, owing to a hard, rigid, and unyielding os, the labor is delayed from hour to hour, a similar administration will immediately produce relaxation, and permit the labor to proceed without interruption. In many forms of dysmenorrhœa its beneficial effects are equally noticeable, but to produce a more satisfactory result in these cases it should be administered for a month or two daily. By keeping the system thus for some time thoroughly under its influence, the pelvic circulation becomes more free, all congestive symptoms seem to disappear, the uterus and its appendages are relaxed, and, being freed from the tense and rigid condition which usually prevails in these conditions, menstruation becomes free and painless.

But I wish more especially to call attention to the action of gelsemium in those diseases of women which have assumed more or less of a chronic form, and in which, from a long continued condition of congestion, irritation, and perhaps inflammation of the pelvic viscera, the nerve centers have become so disturbed that the disease is finally classified under the head of the "neuroses." Possibly no class of patients suffer more from incorrect medication than these. Surely there is none concerning which there is so wide a diversity of opinion regarding the diagnosis of individual cases and the medication to be employed to remove the disease.

After we have read the various conflicting pages of authority on this subject till the mind is confused in the attempt to come to a correct understanding of the pathological conditions which distinguish the various phases of hyperæmia and anæmia of the spinal cord, or the same conditions confined to the anterior or posterior columns and the base of the brain, hysteria, spinal irritation, neurasthenia, and the various mental phenomena which approach the borders of insanity; after we have taken a survey of the entire armamentarium which the medical world has stored up for this purpose, including phosphorus, strychnine, iron, the bromides, electricity, and that much abused nineteenth century method, "local treatment," we are ready to close our eyes. We can easily imagine before us the helpless form of woman, piteously and vainly extending her hands for help. Now, while I do not claim to have discovered a never failing remedy—a panacea—for all these conditions, yet I do hold that a large number of the diseases of females can be relieved, benefited, or cured by this remedy. I reason that in the most of them there is an inefficiency or unequal distribution of nerve force, and consequently an unequal distribution of blood supply. The nerve centers being stimulated to a correct performance of their functions, there follows an equal and normal supply of blood.

Gelsemium has an apparent two-fold action on the spinal cord. When given in minute quantities and long continued, it stimulates, gives tone and elaborates nerve force, while the opposite effect is produced if the dose is sufficiently increased. This apparently dual mode of action may be considered simply as different degrees of the same action, and not as an illustration of the homœopathic idea of infinitesimal action, or a verification of the theory of *similia similibus curantur*. No more rational explanation of this mode of action can be given than that explaining the way in which small doses of ipecac lessen the irritability of the gastric mucous membrane, and in this way check vomiting; or in which strychnia in small quantities is used to control some forms of convulsion. Prof. Ringer has satisfactorily demonstrated this primary stimulating action of the article by noting its effect in some of the lower animals on the third pair of nerves. By a series of carefully conducted experiments, he shows conclusively that whether given internally, or applied topically, contraction of the pupil invariably precedes dilatation, thereby proving that its first effect is that of stimulation. After having administered it for a number of years I am now prepared to positively assert that gelsemium is a stimulant, or perhaps I might say tonic, to the cerebro-spinal system when long continued in small quantities. It has yielded the best results in the nervous diseases of women, in connection with many disarrangements of the pelvic viscera. I am inclined to believe that more of these diseases located in the pelvic cavity are due directly to a depressed condition of nervous force, than is generally admitted by the profession to-day, and instead of the local trouble being the cause of the nervous disturbance, the reverse is true. It is this class of cases in which I would most earnestly recommend the long continued use of gelsemium in minute doses. Perhaps a better illustration of this application of the remedy may be given by reciting the history of three cases:

CASE FIRST.—Mrs. I. S. came under my care five years ago; age 35 years; mother of four children, youngest fourteen years old. When I was called to her, she was confined to her bed, and had not taken a step without assistance for five weeks. She was weak, despondent, very nervous, and subject to severe neuralgic attacks in various parts of the body. Examination revealed a tense, swollen condition of the uterus and also much peri-uterine congestion and inflammation. In the region of the right ovary was a large swelling, which was easily recognizable through the walls of the abdomen, and which her medical attendant had designated as a tumor. Her history was the usual history of such cases. Two years before she had felt that her health was giving way. She was languid, tired in mind and body, and seeking counsel from her physician, was told she had ulceration of the womb and must have local treatment. She had it, changed physicians and had it again, and continued to have the same treatment from each physician to whom she applied, varied slightly according to his fancy, or as to whether his diagnosis was ulceration, endocervicitis, retroflexion, retroversion, ovaritis, or peri-uterine inflammation, spinal irritation, etc. Menstruation had for a number of months been growing less, till now, at each period it amounted to only a few drops, or did not appear at all. There were also slight rheumatic symptoms in various parts of the body. Here was a disturbed condition of the nervous system which I believed could be removed by the administration of gelsemium. I immediately began to give it, my prescription being:

℞ Ex. gelsemii Fl.................................gtt.j
Aquæ.......................................fl ℥ii
Teaspoonful three times a day.

The dose was gradually increased till in a month she was taking one-fourth of a drop, and in two months one-half of a drop three times a day. The patient gained gradually in strength, all of the functions of the body being gradually restored, menstruation increased with each period, and at the end of the sixth month was perfect; and the enlargement in the region of the left ovary had entirely disappeared, as also had all symptoms of uterine congestion and inflammation. In eighteen months after the beginning of the treatment she gave birth to a perfectly healthy child. Since that time her health has been perfect.

CASE SECOND.—Mrs. L. S., aged 45, came under my professional care in 1878. She had been in bad health for ten years. She gave a decidedly neurotic history, several members of the family having had insane symptoms. She herself had frequent attacks of severe neuralgia. She was melancholy, dizzy, felt exhausted on the slightest exertion, and complained of a feeling of numbness over the left lateral half of the body, which was increased at the menstrual period. This condition of hemi-anæsthesia was undoubtedly hysterical, and not due to any cerebral lesion. Ovarian tenderness was more perceptible over the left ovary. The bowels were constipated; micturition was frequent. On walking there was much bearing down in the back and lower abdomen. Examination revealed ovarian tenderness; the uterus appeared enlarged and retroverted. Menstruation had been very irregular for several months, sometimes not appearing for two months. She suffered severely with dysmenorrhœa; had been married twenty years but had never been pregnant. She had received a great deal of local treatment, and worn a number of pessaries of different kinds. Her physician had attributed her entire trouble to ovarian and uterine disease. I began the administration of gelsemium in doses of one-sixteenth of a drop, and gradually increased to half a drop three times a day. Her improvement was gradual but continuous. In three months her strength had much increased. The hemi-anæthæsia had almost disappeared. She was much more cheerful in disposition. Menstruation had appeared regularly, and was entirely painless and much increased in quantity. In six months all symptoms of ovarian or uterine inflammation had entirely disappeared, and she appeared well, with the exception of not having yet arrived at her normal standard of strength. Much exertion produced unnatural fatigue. I advised a continuance of the remedy. About that time she changed her residence to Michigan. In about three months she came across the lake one stormy day, and called on me at my office full of anxiety and fear at what she termed her "strange feelings." Menstruation had not appeared for two months, she was nauseated, vomited frequently, and felt, as she expressed it, "so very strangely," such feelings she had never had before. She was fearful of an attack of paralysis. I made an examination, and found her pregnant. At this her alarm was greatly increased, and she pleaded piteously that I would again establish menstruation, declaring that as she had never been pregnant, and was now 45 years old, it would be certain death for her to attempt to bring a child into the world. I quieted her fears, gave small doses of oxalate of cerium and subnitrate of bismuth, and had her return home and write me every few days her condition. She continued to do well during the entire period of utero-gestation, and on the day of the birth I received a telegram, "wife and son doing well." A few months ago she called on me, and was leading a beautiful five-year-old boy by the hand. This was a remarkable case, not only for the removal of the pathological condition, but that menstruation should return and pregnancy occur at the age of forty-five years.

CASE THIRD.—Mrs. G. B., aged 46, consulted me June 18, 1880. Her case presented a number of interesting features, and was remarkable for the extremely low state to which the general system had been reduced, and the variety of diagnoses which had been given by medical gentlemen. For eight years there had been an apparent decline of health, and at the same time a perceptible loss of flesh, till at the time of her consulting me the weight was reduced to seventy-five and one-half pounds.

Five years previously she had suffered from an attack of typhoid fever of five weeks' duration, and since that time had been treated for sciatica, bronchitis, ulceration of the intestinal canal, disease of the pyloric orifice of stomach, cirrhosis of the liver, diseased kidneys, hyperæmia of the brain, and last but not least, the various flexions, versions, and inflammations of the uterus. She was a lady of unusual intelligence, and beyond the mere desire to recover, manifested a scientific interest in her own case, and had kept a careful record of opinions which had been given her by medical men concerning her condition. This recording of medical opinions seemed to have grown almost to a monomania with her, and no sooner had I finished my examination than she took out pencil and paper, and enquired, "and now, doctor, what is your diagnosis?" I facetiously replied that as there was no room to make a new diagnosis, I would simply say that she was ill. Her case presented symptoms which indicated a general failure of nutrition. As before stated, at the time of beginning treatment she only weighed seventy-five and one-half pounds. Her symptoms were restlessness, inability to sit still, easily worried, frequent attacks of neuralgia, sleeplessness, depression of spirits, pressure on the top of the head, spinal tenderness, more marked over spinal processes of the third and fourth dorsal vertebræ, constipation, and a very feeble condition of digestion, with frequent acid eructations. She complained much of back-ache, and heavy dragging sensation around the loins, but examination revealed but slight engorgement of the uterus and ovaries. Menstruation had been very scant, and irregular for two or three years, and had not appeared since November preceding, seven months before. I immediately recognized in this case a condition of deficient and irregular supply of nerve force, and prescribed:

℞ Ex. gelsem. Fl.......................... .....gtt.j
Aquæ.................................... ℥ii
M. Teaspoonful three (3) times a day.

She began to improve almost immediately. I increased the dose in one month to double the amount. The digestive organs resumed their natural function, the nervous system became quieted, sleep was more prolonged and natural, and at the third month menstruation reappeared, much to her surprise, and somewhat to my own, as she appeared to have approached the menopause. In October, four months from the beginning of the treatment, she weighed ninety-five and one-half pounds, indicating a gain of just twenty pounds. I gradually increased the dose of medicine till I had reached one-fourth of a drop, when the patient declared she felt the effects of each dose in the eyes half an hour after taking, when I reduced the quantity to one-eighth, at which I continued for about four months longer, when her health seemed to be perfect. She removed to the city of Philadelphia, and I did not see her for a year, at which time she called and appeared in excellent condition of mind and body. About four months ago, when passing through the city, she called at my office, and still reported good health. She has had altogether a gain of thirty-five pounds since the beginning of the treatment, and menstruation still continues at the age of almost fifty years.

These cases are illustrative of a large number in which I have used gelsemium in small doses, with equally beneficial results. Whether the influence this remedy has over the pelvic viscera is due directly to an impression on the spinal cord, or upon the sympathetic ganglia, is a matter of some question; but it is highly probable that it has a tonic effect on the sympathetic ganglia as well as the cerebro-spinal centers. The action of gelsemium over the disorders peculiar to woman, I regard as a subject of great importance, and well worthy the attention of the profession. On no subject of diseases is there so great a diversity of opinion as here, and in no branch of medicine is there so great a demand for improvement in therapeutics. We bow with respect in the presence of those who in past years have formulated theories in regard to these diseases. Each age can read only by the light admitted to the altitude at which it stands. Looking back over the history of gynæcology with all the light of to-day and yesterday thrown upon it, can we refuse to acknowledge that a large number—a very large number—of the diseases in its field arise from a disturbed or depressed condition of the nervous system? We are living in a nervous age, and our spurious civilization rests heavily upon us. Dr. T. Gaillard Thomas, in speaking of woman and her diseases says, "The customs of civilized life have depreciated her powers of endurance and capacity for resisting disease." In the year 1850, Dr. Tilt, of London, affirmed that "as a rule, pelvic diseases of women radiate from morbid ovulation." Can it be wondered at, when the nervous system, the seat of nearly all the vital forces, is shattered, that nature should make an attempt to cut off the supply of sickly offspring by disturbing ovulation? This position of Dr. Tilt has been denied by many, but undoubtedly it contains the germs of truth. To-day I would go beyond this, and say that a large number—a very large number—of the pelvic disorders of women arise from a disturbed condition of the nervous system, which in turn produces morbid ovulation. It is in this way that I account for the action of gelsemium over these disorders. Its action is directly on the nerve centers—spinal and sympathetic, equalizing the circulation, giving tone to cell-power, by these means permitting free ovulation, and so relieving the congested and inflamed condition of the other pelvic viscera. If this be true, if it does have the action illustrated by the cases I have cited, I propound the question, may it not also be considered a remedy for sterility?

---

### A NEW METHOD IN ADHERENT PLACENTA.

The New York *Medical Record*, May 17, 1884, alludes to a report in the *Clinique*, by Dr. J. Feld, of Kansas City, on "A New Method in Adherent Placenta." In six cases of adherent placenta, the life of the woman was saved by pumping cold water through the umbilical cord.

Although the *Record* is profoundly in erroi, in its denomination of the method as a new one, it deserves credit for calling attention to a very old and very excellent procedure. The injection of cold water into the umbilical vein, in *post-partum* hæmorrhage and adherent placenta, was ably advocated and extensively practiced by Mojon (1826), Kilian, and others.

The umbilical vein is divided transversely, a tube or quill fastened securely in the proximal extremity, and cold water injected, slowly and carefully, into the placenta, by means of an ordinary syringe. The cold water, forcing its way into the placenta, distends that organ to twice its original volume, escaping through the lacerated utero-placental vessels, bathes the endometrium, and stimulates the uterus to powerful contractions, usually resulting in the total separation and spontaneous expulsion of the afterbirth. Stoltz and Rombach (1855) have extolled most highly this measure, while Linéard (1875) claimed that the injection of 150 grammes of pure cold water into the umbilical vein was "the best, most reliable, and least dangerous expedient in all cases of *post-partum* hæmorrhage, or adherent placenta."

At present this method is extensively practiced in Germany, more particularly in Bavaria. Scanzoni, in Würzburg, practices this method exclusively. W. W. JAGGARD, M. D.

May 26, 1884.

